# Administration of imatinib after allogeneic hematopoietic stem cell transplantation may improve disease-free survival for patients with Philadelphia chromosome-positive acute lymphobla stic leukemia

**DOI:** 10.1186/1756-8722-5-29

**Published:** 2012-06-08

**Authors:** Huan Chen, Kai-yan Liu, Lan-ping Xu, Dai-hong Liu, Yu-hong Chen, Xiang-yu Zhao, Wei Han, Xiao-hui Zhang, Yu Wang, Yuan-yuan Zhang, Ya-zhen Qin, Yan-rong Liu, Xiao-jun Huang

**Affiliations:** 1 Peking University People’s Hospital, Peking University Institute of Hematology, Beijing Key Laboratory of Hematopoietic Stem Cell Transplantation, Beijing, 100044, P.R. China; 2 Peking University People’s Hospital, Peking University Institute of Hematology, No.11, Xizhimen South Street, Xicheng District, Beijing, 100044, P.R. China

**Keywords:** Philadelphia chromosome, Acute lymphoblastic leukemia, Allogeneic hematopoietic cell transplantation, Minimal residual disease, Imatinib

## Abstract

**Background:**

Maintenance therapy with imatinib during the post-transplant period has been used for patients with Philadelphia chromosome-positive acute lymphoblastic leukemia (Ph + ALL); however, its efficacy has not been demonstrated. A study was designed to investigate the safety of imatinib and its efficacy in preventing hematological relapse and improving disease-free survival (DFS) when administered after allogeneic hematopoietic stem cell transplantation (allo-HCT).

**Methods:**

Patients with Ph + ALL that received allo-HCT were enrolled in the study. Real-time quantitative reverse-transcription polymerase chain reaction (qRT-PCR) was used to detect *BCR-ABL* transcript levels. Imatinib therapy was initiated if patient neutrophil counts were > 1.0 × 10^9^/L and platelet counts were > 50.0 × 10^9^/L, or if they displayed either elevated *BCR-ABL* transcript levels in two consecutive tests, or a *BCR-ABL* transcript level ≥ 10^-2^ after initial engraftment. Patients receiving imatinib after relapse were assigned to the non-imatinib group. The imatinib treatment was scheduled for 3–12 months, until *BCR-ABL* transcript levels were negative at least for three consecutive tests or complete molecular remission was sustained for at least 3 months.

**Results:**

A total of 82 patients were enrolled. Sixty-two patients initiated imatinib therapy post-HCT. Imatinib therapy was initiated at a median time of 70 days post-HCT. Grade 3–4 adverse events (AEs) occurred in 17.7% of patients. Ten patients (16.1%) terminated imatinib therapy owing to AEs. Among the patients in imatinib and non-imatinib groups, the estimated 5-year relapse rate was 10.2% and 33.1% (p = 0.016), and the 5-year probability of DFS was 81.5% and 33.5% (p = 0.000) with the median follow-up of 31 months (range, 2.5-76 months) and 24.5 months (range, 4–72 months), respectively. Multivariate analysis identified imatinib maintenance therapy post-HCT as an independent prognostic factor for DFS (p = 0.000, hazard ratio [HR] =4.8) and OS (p = 0.000, HR = 6.2).

**Conclusions:**

These results indicate that relapse rate can be reduced and DFS may be improved in Ph + ALL patients with imatinib maintenance therapy after HCT. *BCR-ABL*monitoring by qRT-PCR can guide maintenance therapy with imatinib including initiation time and treatment duration after allo-HCT.

## Introduction

Allogeneic hematopoietic stem cell transplantation (allo-HCT) is still considered the optimal curative treatment for Philadelphia chromosome-positive acute lymphoblastic leukemia (Ph + ALL). The disease-free survival (DFS) of Ph + ALL patients after allo-HCT ranges from 21% to 57% [1-4]. The main cause of treatment failure is relapse, and approximately 30% of patients that undergo allo-HCT in first complete remission (CR_1_) eventually relapse. Of the patients that undergo allo-HCT beyond CR_1_, or for those with refractory disease, the relapse rate is even higher. The tyrosine kinase inhibitor, imatinib, has been widely used for the treatment of chronic myelogenous leukemia [5,6], and has recently been used for treatment of Ph + ALL. Since the introduction of imatinib in the combination chemotherapy regimes for newly diagnosed Ph + ALL, more than 95% of patients can achieve CR_1_. Several studies have shown decreased relapse rates and improved DFS for patients with imatinib-based treatment prior to allo-HCT, compared with their historical controls [7-9]. However, the efficacy of maintenance therapy with imatinib after transplant for Ph + ALL patients is still uncertain.

Detection of minimal residual disease (MRD) after transplant is associated with an increased risk of relapse [10]. An early study by Wassmann *et al* showed that MRD-triggered imatinib therapy led to complete molecular remission (CR^mol^) in 52% of patients expressing *BCR-ABL* after HCT; however, approximately 50% of patients ultimately experienced hematological relapse [11]. In addition, it was reported that 23% of Ph + ALL patients that screened negative for *BCR-ABL* after allo-HCT relapsed [12]. Thus, earlier initiation of imatinib treatment in the setting of low leukemia burden, or negative detection of MRD after HCT, may reduce the relapse rate and improve survival to an even greater extent. The feasibility and safety of early prophylactic administration of imatinib after HCT has been previously confirmed [13]. However, imatinib toxicity is relevant when started soon after HCT.

We previously demonstrated that administration of imatinib in the first 90 days after allo-HCT, based on MRD monitoring, is feasible, and the toxicity is acceptable. Preliminary results showed that treatment outcome was significantly improved compared with our previous study [4]. In this phase II study, we evaluated the safety and efficacy of imatinib therapy, when initiating treatment based on patient clinical conditions and *BCR-ABL* transcript levels after allo-HCT. We also investigated the factors that may impact relapse and survival.

## Materials and methods

### Patient eligibility

Allo-HCT recipients diagnosed with Ph + ALL (< 60 years of age) were eligible for the study, regardless of the source of HCT (from either HLA-matched sibling donors, unrelated donors or mismatched related donors). The diagnosis of Ph + ALL was based on the WHO diagnosis criteria. Patients were excluded from the study if they displayed hypersensitivity or were assessed as resistant to imatinib before HCT. Patients were also excluded if either hematological relapse or extramedullary leukemia involvement was diagnosed after initial engraftment, or if the life expectancy was less than 1 month post-HCT. The study was reviewed and approved by the ethics committee at Peking University People’s Hospital. All patients provided written, informed consent before transplantation.

### Conditioning regimen and graft-versus-host disease (GVHD) prophylaxis

All patients received a myeloablative transplant. Conditioning regimens were as previously described [14,15]. In matched sibling transplants, the conditionings were (1) total body irradiation (TBI) with 7.7-12.0 Gy and cyclophosphamide (Cy) 1.8 g/m^2^/d × 2 days or (2) hydroxyurea (40 mg/kg, q12 h) given on day −10, cytosine arabinoside (Ara-C, 2 g/m^2^/d) intravenously on day −9; busulfan (Bu,3.2 mg/kg per day) intravenously on days −8 to −6; Cy (1.8 g/m^2^/d) intravenously on days −5 and −4; Methyl-N-(2-chloroethyl)-N-cyclohexyl-N-nitrosourea (Me-CCNU, 250 mg/kg/d) orally once on day −3. Patients that underwent mismatched related and unrelated HCT were given cytosine arabinoside (2–4 g/m^2^/d) on days −10 and −9 in the Bu/Cy regimen (as shown above) and anti-human thymocyte globulin (ATG, 2.5 mg/kg/d,Sang Stat, Lyon, France) intravenously for 4 consecutive days from days −5 to −2.

All transplant recipients received cyclosporin A-based acute GVHD prophylaxis [14,15]. Supportive care was administered as previously described [14].

### MRD assessment

The level of *BCR-ABL* transcripts in patient bone marrow was assessed by TaqMan-based real-time quantitative reverse-transcription polymerase chain reaction (qRT-PCR), as previously described [16]. The *BCR-ABL* primers and probe that amplify both the *b3a2* and *b2a2* junctions are previously published [17]. The primers and probe that amplify the *ABL* and *e1a2 BCR-ABL* junctions are listed in the report of the Europe against Cancer Program [17,18]. *BCR-ABL* transcript level was calculated as: fusion transcript copies / *ABL* transcript copies × 100 (%). The *ABL* copy number of all the samples included in this study was greater than 3 × 10^4^. The reproducible sensitivity of qRT-PCR was five copies. A CR^mol^ was defined as the negative expression of *BCR-ABL* by qRT-PCR in patient bone marrow specimens. Bone marrow aspiration for morphological and cytogenetic analysis (fluorescence *in situ* hybridization, FISH), flow cytometry and qRT-PCR was scheduled for 1, 3, 6, 9 and 12 months post-HCT (qRT-PCR tests were repeated at 2-week intervals if necessary), then once every 6 months between months 12 and 24 post-HCT.

### Study design and treatment

Treatment with imatinib was initiated: (1) if patient peripheral blood absolute neutrophil counts (ANC) were >1.0 × 10^9^/L without granulocyte colony-stimulating factor (G-CSF) administration, and the platelet count was >50.0 × 10^9^/L, regardless of the level of *BCR-ABL* transcript; or (2) if the level of *BCR-ABL* transcript in the bone marrow was detectable and transcript levels increased for two consecutive tests, or if the *BCR-ABL* transcript level was ≥10^-2^ after the initial engraftment, although patients ANC or platelet count were below the above values. Other criteria for initiation of treatment included that patients could tolerate oral imatinib without gut GVHD or life-threatening infection.

Imatinib treatment was scheduled for 3–12 months after HCT, until *BCR-ABL* transcript levels were negative at least for 3 consecutive tests or CR^mol^ was sustained for at least 3 months, as described in our previous report [19].

The initial dose of imatinib was 400 mg/d for adults (> 17 years) and 260 mg/m^2^/d for children (< 17 years). The daily dose of imatinib was adjusted according to the National Comprehensive Cancer Network practice guideline regarding the management of imatinib toxicity (version 2005). The dose of imatinib was reduced to 300 mg/d if the ANC was <1.0 × 10^9^/L, despite administration of G-CSF, or if the platelet count was less than 20 × 10^9^/L. The dose of imatinib could escalate to 600 mg/d (340 mg/m^2^/d in children <17 years). The minimum acceptable dose of imatinib was 300 mg/d (260 mg/m^2^/d for children <17 years) for at least 5 days per week.

Patients were permitted to voluntarily withdraw from the study at any time or were withdrawn if grade 3 or 4 toxicity was sustained for more than 2 weeks, despite interrupting the imatinib therapy.

### Safety and efficacy

The primary study end point addressed patient safety. The toxicity of imatinib was assessed according to the Common Toxicity Criteria, version 3.0. The secondary study endpoint assessed the efficacy of imatinib therapy. The efficacy evaluation included relapse rate, DFS and overall survival (OS). A post-transplant relapse was defined as hematological relapse, extramedullary involvement of leukemia and cytogenetic relapse. DFS was defined as continuous survival without relapse or death from any cause after HCT. OS was defined as continuous survival until death from any cause after HCT. Patients who were treated with imatinib for less than 7 days were not included for the efficacy evaluation, but were included in the safety evaluation.

### Statistical analysis

Parametric tests used the χ^2^ test or Fisher’s exact test. The Mann–Whitney U test was used for nonparametric tests. Univariate analysis for DFS and OS of all enrolled patients was conducted using Kaplan-Meier analysis with the log-rank test. The factors included in the univariate analysis were sex, age (> 30/< 30 years), disease status pre-HCT (CR_1_/> CR_1_), *BCR-ABL* transcript levels before and after HCT, donor type, acute and chronic GVHD, imatinib therapy versus no treatment, post-HCT. Multiple regression analysis for DFS and OS was conducted using multiple Cox regression. The covariates adjusted in the multiple regression models included factors identified as significant in the univariate analysis (p < 0.05). Kaplan-Meier analysis was used to estimate DFS and OS, while cumulative incidence was calculated for non-relapse mortality (NRM) and relapse rate. The relapse rate was also calculated by taking into account the competing risk of death due to other complications using the Fine-Gray model. The log-rank test was used to compare the survival curve and the Gray test for cumulative incidence curve between the imatinib and non-imatinib groups. Data analysis was performed using the SPSS and R software packages, and a p-value < 0.05 was considered statistically significant.

## Results

### Patient enrollment and engraftment

From May 2005 to March 2010, 82 patients (median age, 28.5 years; range, 3–51 years) consented to participate in the study. The demographic characteristics and relevant transplantation data for these individuals are shown in Table 1. Imatinib therapy was initiated in 62 patients, according to the study regimen. Within this group, two patients received imatinib therapy for less than 7 days, owing to severe gut GVHD and grade 3 hematologic toxicity, respectively, and thereafter were not included in the efficacy evaluation. Twenty patients did not receive imatinib therapy for the following reasons: pancytopenia (n = 2), severe infections (n = 6), severe gut GVHD (n = 7) or personal decisions (n = 5).

**Table 1:** Patient characteristics in the imatinib and non-imatinib groups

	**Imatinib group**	**Non-imatinib group**	**P-value**
Number of patients	62(75.6%)	20(24.4%)	
Age (y), median (range)	29(6-50)	27.5(3-51)	0.256
<30 / >30 y	37/25	10/10	0.604
Sex (M/F)	43/19	12/8	0.071
Disease status pre-HCT			
CR1/ >CR1	50/12	16/4	1.000
Additional cytogenetic			
abnormality			
Yes/No	3/59	1/19	0.272
Imatinib therapy before HCT			
Yes/ No	54/8	14/6	0.180
RT-PCR-BCR/ABL(pre-HCT)			
0/ >0	26/36	6/14	0.752
Donor type			
HLA matched siblings	15	4	0.199
MMR	45	15	
MUD	2	1	
Stem-cell source			
PBSCT	7	2	1.000
BMT + PBSCT	55	18	
Conditioning regimen			
BUCY	62	17	0.013
TBI/CY	0	3	
Engraftment			
Myeloid (day),median(range)	13(10-22)	13(10-27)	0.248
Platelet (day),median(range)	17(9-150)	13(7-30)	0.268
RT-PCR-BCR/ABL(post-HCT)			
0/ >0	48/ 14	18/ 2	0.540
Acute GVHD			
0/ I°-II°/ III-IV°	30/ 28/ 4	5/ 11/ 4	0.199
Chronic GVHD			
no/ L/ E-cGVHD	22/ 25/ 12	3/ 8/ 8	0.223

CR1, first complete remission; MMR, mismatched related; MUD, matched unrelated donor; BMT, bone marrow transplant; PBSCT, peripheral blood stem cell transplant; L, local; E-cGVHD, extensive chronic GVHD.

All 82 patients achieved myeloid engraftment after HCT. All patients, except one, achieved platelet engraftment. The engraftment time was not significantly different between the imatinib and non-imatinib groups (Table 1).

### Imatinib treatment and safety

All enrolled patients achieved hematological remission and all of them were in complete cytogenetic remission (CCyR) following myeloid engraftment. Treatment with imatinib was initiated at a median time of 70 days post-HCT (range, 20–270 days), and the median duration of imatinib therapy was 90 days (range, 13–540 days).

Thirty-four adult patients tolerated imatinib at a daily dose of 400 mg. The daily dose of imatinib was decreased to 300 mg in 16 adults for 5–7 days a week. Seven out of 10 children tolerated imatinib at a daily dose of 260 mg/m^2^.

Sixty-two patients were included in the safety evaluation, including both hematological and non-hematological toxicities (Table 2). Of these, 44 patients (70.9%) experienced different adverse events (AEs) that were possibly due to imatinib therapy. Two, simultaneous AEs were observed in 28 patients. Grade 3–4 AEs occurred in 11 (17.7%) patients, and were all reversible. Ten patients (16.1%) treated with imatinib for less than 3 months, terminated treatment because of AEs (n = 9) or gut GVHD (n = 1).

**Table 2:** Number of patients with adverse events related to imatinib

**Event Classification**	**No. of patients with AEs**				
		**0**	**1-2**	**3**	**4**
Hematology	36	26	32	3	1
Neutropenia	36	26	32	2	2
Thrombocytopenia	36	26	34	1	1
Edema	19	43	16	3	0
Nausea/emesis	29	33	24	4	1
Elevation of ALT/AST	3	59	3	0	0

AEs, adverse events.

### Response to imatinib therapy and outcome after HCT

After HCT, the bcr-abl tests by qRT-PCR were negative in 48 patients prior to administration of imatinib. Within this group, hematological relapse occurred in 4 patients (1 received imatinib for less than 7 days after HCT) and extramedullary leukemia relapse occurred in one patient. These five patients received other treatment option in addition to imatinib, including chemotherapy or irradiation, followed by donor lymphocyte infusion (DLI). Of this group, two patients died from relapse and two patients died from non-relapse complications.

Prior to imatinib therapy, 14 patients tested positive for *BCR-ABL* expression following engraftment. Eight patients became *BCR-ABL* negative 1 month after imatinib therapy (range, 1–3 months). Two patients died from hematological relapse. Four patients displayed persistent *BCR-ABL* expression for 3 months and achieved CR^mol^ with additional treatment such as chemotherapy plus DLI, second-generation tyrosine kinase inhibitors, or secondary allo-HCT. Four patients died from non-relapse complications, including respiratory failure (n = 2); demyelinating polyneuropathy (n = 1) or post-transplant lymphoproliferative disease (n = 1).

In the non-imatinib treated group, five patients died from hematological relapse. One patient with central nervous system relapse survived in complete remission after receiving central nervous system irradiation and imatinib therapy. The causes of non-relapse death included GVHD (n = 1), infection (n = 3) and others (n = 3).

### Relapse rates and NRM

**Figure 1:**
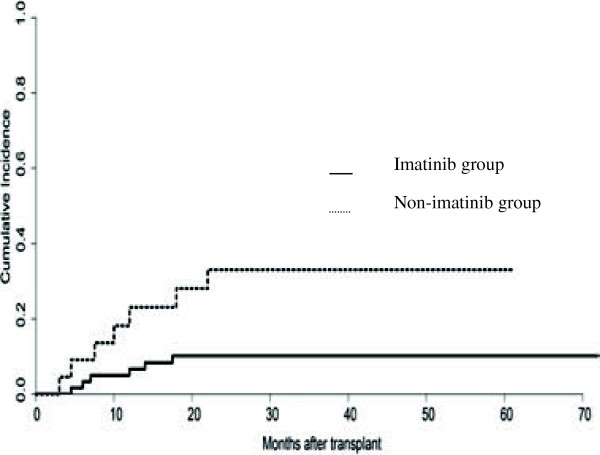
**Cumulative incidence of relapse for patients in imatinib and non-imatinib groups, post-HCT.** The relapse rate was calculated by taking into account the competing risk of death due to other complications. The cumulative incidence of relapse was significantly lower in imatinib compared with non-imatinib treated groups (10.18% *vs* 33.05%, p = 0.016).

At the time of the last follow-up (November 2011), a total of 13 patients had relapsed, including hematological relapse (n = 11) or extramedullary leukemia relapse (n = 2). No cytogenetic relapse occurred without hematological relapse concurrently. The overall 5-year relapse rate was 18.34 ± 4.8%. The estimated 5-year probability of relapse between the imatinib and non-imatinib treated groups was 10.2% ± 3.9% and 33.1% ± 10.8% (p = 0.016), respectively (Figure 1). The median time of relapse was 9.5 months (range, 2.5-17.5 months) in the imatinib group of patients, post-HCT. The median time of relapse was 12 months (range, 3–22 months) in the non-imatinib group of patients. Three patients experienced molecular relapse at 6, 12 and 18 months after terminating imatinib treatment. All three patients achieved a second CR^mol^ following re-administration of imatinib for 1 month. The NRM among the imatinib group and non-imatinib group was 6.66% ± 3.24% and 37.19% ± 10.89% (p = 0.0006), respectively.

### DFS and OS

**Figure 2:**
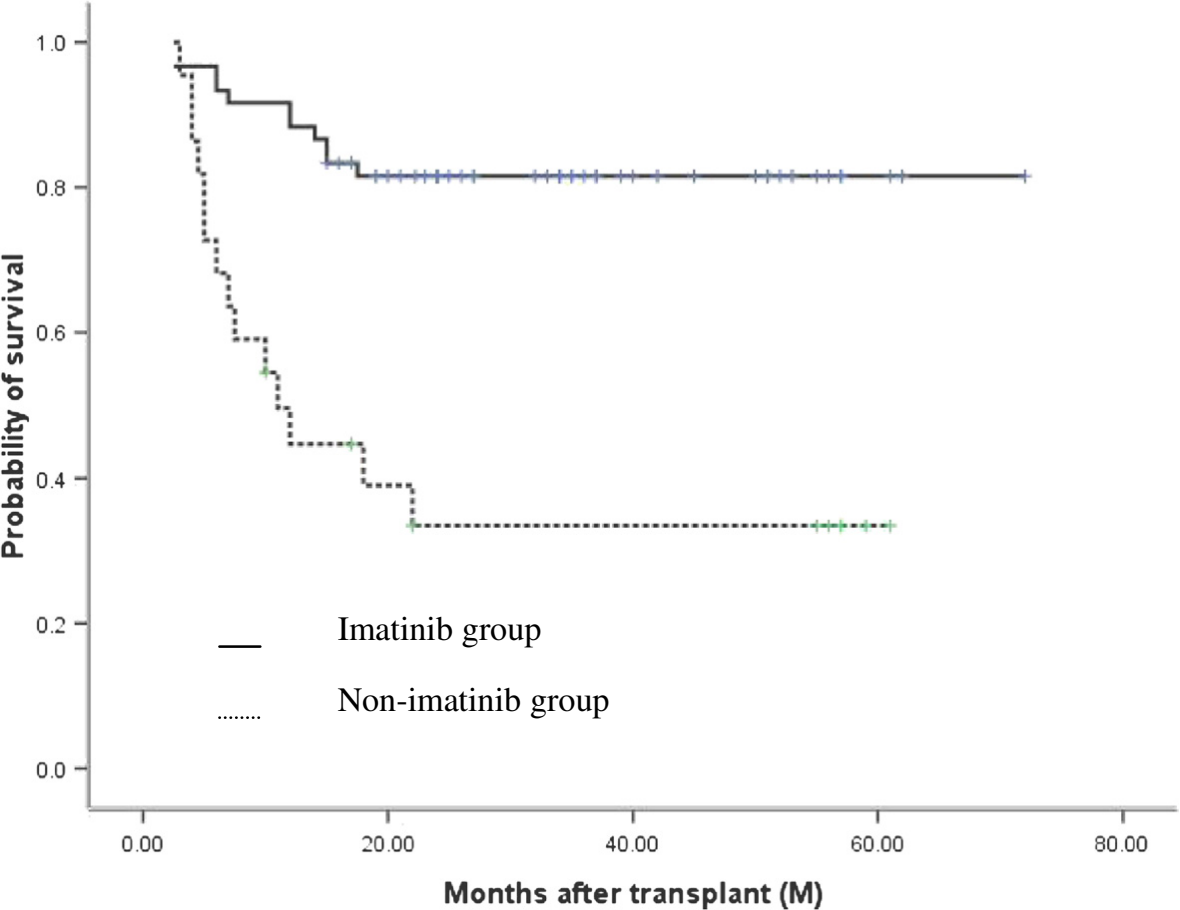
**Disease-free survival (DFS) at 5 years in imatinib and non-imatinib groups, post-HCT.** Kaplan-Meier analysis showed that the 5-year DFS of patients in the imatinib-group was significantly higher than in the non-imatinib group (81.5% *vs* 33.5%, p = 0.000).

**Figure 3:**
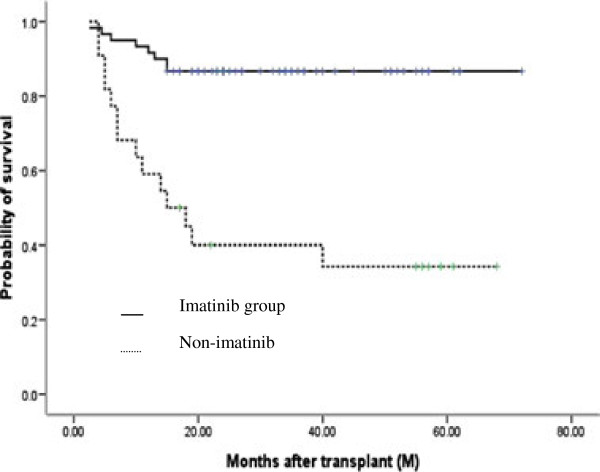
**Overall survival (OS) at 5 years in imatinib and non-imatinib groups, post-HCT.** Kaplan-Meier analysis showed that the 5-year OS of patients in the imatinib-group was significantly higher than the patients in the non-imatinib group (86.7% *vs* 34.3%, p = 0.000).

At the time of the last follow-up (November 2011), 52/62 patients in the imatinib group were alive at a median follow-up of 31 months (range, 2.5-76 months). All patients were in CR^mol^ without imatinib administration. The median time of discontinuation of imatinib therapy was 26 months. In contrast, only 8/20 patients in the non-imatinib group were alive at a median follow-up of 24.5 months (range, 4–72 months). The overall 5-year DFS and OS for all patients was 68.9% ± 5.2% and 71.0% ± 5.6%, respectively. The estimated probabilities of DFS and OS at 5 years was 81.5% ± 5.0% and 86.7% ± 4.4% for the imatinib group, respectively, compared with 33.5% ± 10.6% and 34.3% ± 10.5% for the non-imatinib group (p = 0.000 and 0.000) (Figures 2 and 3).

### Prognostic factors associated with DFS and OS

Univariate analysis revealed that post-HCT maintenance therapy with imatinib treatment, pre-HCT disease status and pre-HCT *BCR-ABL* transcript levels were significant factors impacting DFS and OS (p = 0.000, 0.013, 0.020, respectively).

By multivariate Cox regression analysis, post-HCT imatinib maintenance therapy was identified as an independent prognostic factor for DFS and OS (Table 3).

**Table 3:** Multivariate analysis of factors associated with DFS and OS

**Variable**	**DFS**			**OS**		
	**HR**	**95% CI**	**P**	**HR**	**95% CI**	**P**
non-IM use post-HCT	4.8	2.2-10.8	.000	6.2	2.6-15.0	.000
> CR1 pre-HCT				2.7	1.1-6.6	.023
BCR-ABL(+) pre-HCT	3.7	1.3-10.5	0.014			

IM, imatinib; HR, hazard ratio; CI, confidence interval.

## Discussion

Until now, there have been no large, controlled studies demonstrating that imatinib therapy after HCT can improve DFS. A small non-randomized, single-center study from Minnesota identified a trend toward decreased relapse rate in patients treated with imatinib in the pre- and/or post-transplant period [20]. However, only two patients in their study were treated with imatinib maintenance therapy post-transplant. The reports from the Children’s Oncology Group recently showed that patients receiving imatinib therapy for 6 months following matched sibling donor HCT (n = 19) showed no advantage in 3-year event-free survival (EFS) compared with bone marrow transplantation (BMT) alone [21,22].

We administered imatinib maintenance therapy for Ph + ALL patients after HCT based on patient clinical conditions and *BCR-ABL* transcript levels. Our study demonstrates for the first time that patients treated with imatinib maintenance therapy post-HCT have a lower relapse rate and a survival advantage in term of DFS and OS, compared with non-imatinib treated patients. The limitation to this study was that patients in our trial were not randomized to receive imatinib therapy post-HCT. In addition, more patients died from non-relapse complications in the non-imatinib group compared with the imatinib treated group, which may impact the outcome. It should be noted, however, that the demographic characteristics and certain relevant transplant data were similar between the two patient groups (except for 3 patients receiving TBI/Cy as conditioning regimen in the non-imatinib group), thus allowing for a comparison. Multivariate analysis of all enrolled patients also showed that imatinib maintenance therapy post-HCT was an independent prognostic factor for DFS. Additional carefully designed or randomized studies with large patient cohorts are required, however, to confirm the efficacy of this strategy.

The optimal time for initiating imatinib treatment post-HCT is not well established. Previous studies have shown that the ability of patients to tolerate imatinib therapy decreases in cases of poor engraftment and GVHD reactions following HCT. Early initiation of imatinib is frequently associated with grade 3 or 4 cytopenia in the first 100 days after allo-HCT [23]. A study in which all patients were anticipated to begin imatinib treatment (400 mg/day) from the time of full hematological recovery after HCT showed that 12 of 21 patients initiated imatinib at a median time of 3.9 months post-HCT; however, treatment was interrupted in 10 patients owing to complications such as GVHD [24]. Thus, early initiation of imatinib treatment in patients, regardless of their clinical conditions following allo-HCT, may be limited by transplant-related complications and drug toxicity. A recent multi-center, randomized trial by Pfeifer *et al* revealed no significant difference in OS between patients with pre-emptive imatinib therapy and those with prophylactic administration of the drug after HCT [25]. In this study, more than half of enrolled patients discontinued imatinib therapy in both groups of patients, predominantly owing to gastrointestinal toxicities [25]. These data suggest that there are still limitations in the initiation of imatinib therapy just based on post-HCT *BCR-ABL* transcript levels. Furthermore, imatinib therapy may not need to be initiated at the same time period after HCT in patients with negativity for *BCR-ABL* expression. This report also showed that detection of *BCR-ABL* transcripts within 100 days of transplant is associated with a significantly inferior EFS, despite rapid initiation of imatinib treatment [25]. This indicates that delayed initiation of imatinib therapy after HCT may decrease the efficacy of imatinib therapy for some patients.

Our trial was designed to initiate imatinib therapy based on patient *BCR-ABL* transcript levels, while concurrently taking into account the clinical conditions of individual patients (including blood cell counts, GVHD). Grade 3–4 AEs and interruption of imatinib therapy due to AEs or gut GVHD were relatively low compared with other reports [23-25]. Therefore, we conclude that our treatment strategy balanced the safety and efficacy of imatinib therapy after allo-HCT.

MRD positivity pre- and post-HCT is reported to be associated with a high relapse rate after HCT [10,12]. In our study, we found that detection of *BCR-ABL* expression pre-HCT had a significant adverse impact on DFS. This is in line with recent studies showing that MRD levels at different time points prior to HCT have prognostic relevance, and that lower levels of MRD prior to HCT are associated with better DFS following allo-HCT [26,27].

To date, there is no defined period of administering imatinib therapy post-HCT that has been demonstrated to be more appropriate for reducing relapse rate and improving survival. Most studies suggest arbitrarily using imatinib therapy for 6 months to 1 year after allo-HCT, or for 1 year after the first documentation of *BCR-ABL* negativity post-HCT [11,22]. However, in these studies patients still experienced molecular relapse or even hematological relapse after termination of imatinib treatment. Sustained CR^mol^ is defined by some researchers as *BCR-ABL* negativity lasting for a period of at least 3 months [28]. Our regimen was designed to use imatinib until *BCR-ABL* transcripts were negative at least for 3 consecutive tests or CR^mol^ was sustained for at least 3 months. Our preliminary results showed that the relapse rate was lower and DFS was higher compared with our previous studies (DFS, 37.1%) [4]. These data support the rationale of our strategy, which employs regular monitoring of *BCR-ABL* transcript levels by qRT-PCR to guide the treatment period in which imatinib therapy should be administered after HCT. Other factors such as graft-versus leukemia effect may also contribute to eradicating MRD when combined with imatinib therapy after allogeneic transplant. We are aware, however, that the patient numbers in our study are still limited, and future studies involving larger patient cohorts with a longer follow-up period are needed to accurately define the time period of imatinib therapy post-HCT.

Our study also showed that the remission status at the time of HCT significantly predicted OS. Thus, patients transplanted in CR_1_ had significantly higher OS rates compared with those transplanted in > CR_1_. These data are supported by other studies involving both matched-related and unrelated donors for both adults and children with either Ph-ALL or Ph + ALL [29-31]. Therefore, we still recommend that patients with Ph + ALL undergo allo-HCT in CR_1_ if they have available donors.

## Conclusions

In summary, our study demonstrates that relapse rate can be reduced and DFS may be improved in Ph + ALL patients using imatinib maintenance therapy. *BCR-ABL* monitoring by qRT-PCR can well guide imatinib therapy including initiation time and duration of treatment after HCT. Even in the imatinib era, Ph + ALL patients with available donors will benefit from receiving allo-HCT in CR_1_.

## Abbreviations

Ph + ALL, Philadelphia chromosome acute lymphoblastic leukemia; allo-HCT, Allogeneic hematopoietic cell transplantation; qRT-PCR, Quantitative reverse-transcription polymerase chain reaction; DFS, Disease-free survival; OS, Overall survival; ANC, Absolute neutrophil count; MRD, Minimal residual disease; TKI, Tyrosine kinase inhibitor; CR1, First complete remission; CRmol, Complete molecular remission; TBI, Total body irradiation; G-CSF, Granulocyte colony-stimulating factor; NRM, Non-relapse mortality; AEs, Adverse events.

## Competing interests

The authors declare no competing financial interests.

## Authors’ contributions

H.C. designed the research, interpreted the data and wrote the manuscript; K.-Y.L., X.-L.X., D.-H.L., Y.-H.C., X.-Y.Z., W.H., X.-H.Z., Y.W., and Y.-Y.Z. performed the study and contributed to writing the manuscript; Y.-Z.Q. and Y.-R.L. performed the *BCR-ABL* RT-PCR and flow cytometry assays. X.-J.H. is the principal investigator, designed the research, interpreted the data, and wrote the manuscript. All authors read and approved the final manuscript.
